# Supplementary material for: Towards artificial general intelligence via a multimodal foundation model
Source: Nat Commun. 2022 Jun 2;13:3094. doi: 10.1038/s41467-022-30761-2 (PMC9163040; doi:10.1038/s41467-022-30761-2)
Supplement: Supplementary file 1 — Supplementary Information [file 41467_2022_30761_MOESM1_ESM.pdf]

Towards artificial general intelligence via a multimodal foundation model – supplementary note

Nanyi Fei[1,2,3], Zhiwu Lu[1,2], Yizhao Gao[1,2], Guoxing Yang[1,2], Yuqi Huo[2,3], Jingyuan Wen[1,2], Haoyu Lu[1,2], Ruihua Song[1,2], Xin Gao[4], Tao Xiang[5], Hao Sun[1,2] and Ji-Rong Wen[1,2,3]

[1] *Gaoling School of Artificial Intelligence, Renmin University of China, Beijing, China*
[2] *Beijing Key Laboratory of Big Data Management and Analysis Methods, Beijing, China*
[3] *School of Information, Renmin University of China, Beijing, China*
[4] *Computer, Electrical and Mathematical Sciences and Engineering Division, King Abdullah University of Science and Technology, Thuwal, Saudi Arabia*
[5] *Department of Electrical and Electronic Engineering, University of Surrey, Guildford, United Kingdom*

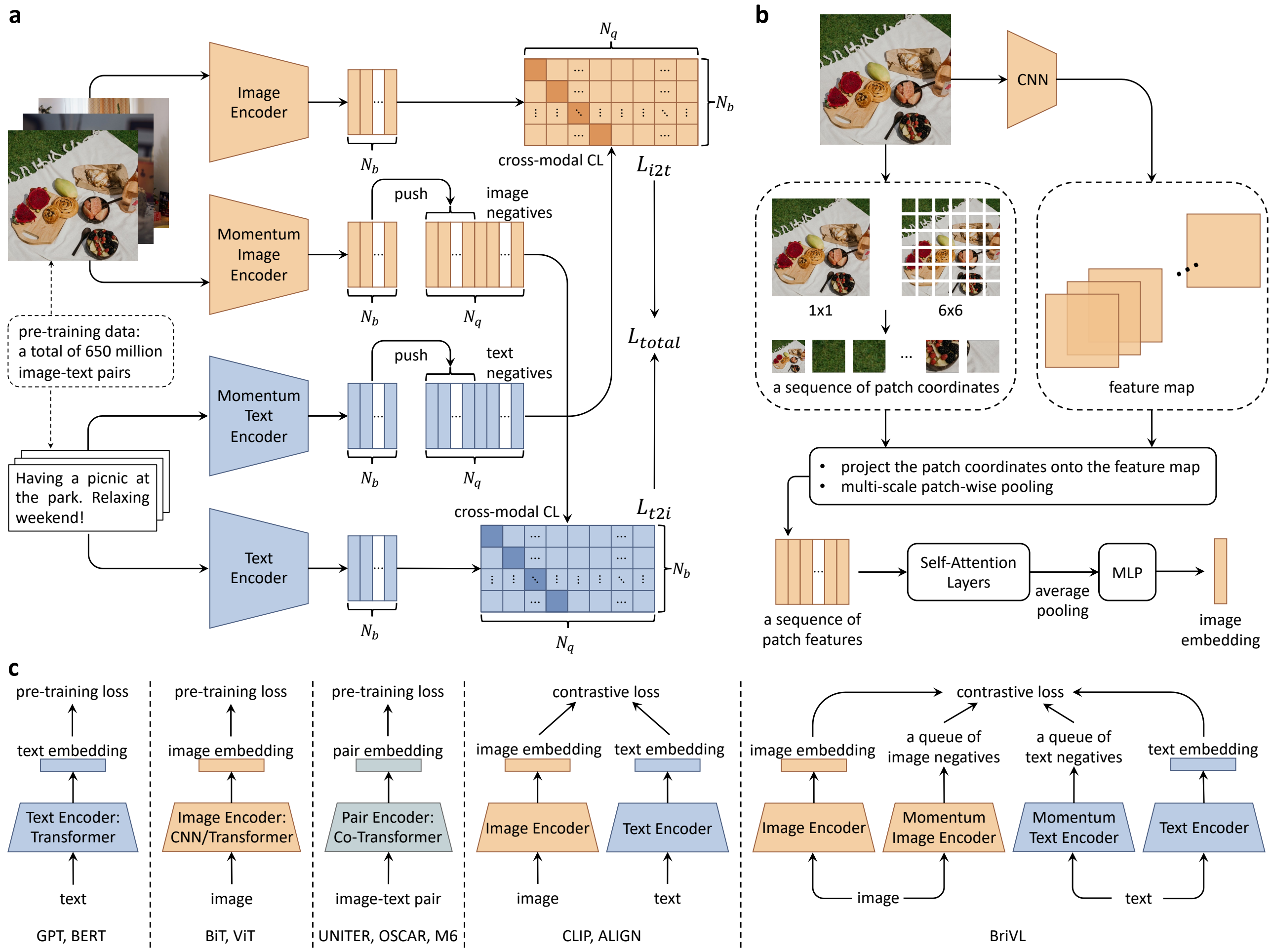

Fig. S1: **Illustrations of our algorithm design, our network design, and existing pre-training architectures.** **a**. The schematic illustration of the proposed BriVL model for large-scale multimodal pre-training. **b**. The detailed network architecture of our image encoder in BriVL. **c**. Illustration of existing pre-training architectures. From left to right: single-modal textual pre-training (e.g., GPT [1] and BERT [2]); single-modal visual pre-training (e.g., BiT [3] and ViT [4]); single-tower multimodal pre-training (e.g., UNITER [5], OSCAR [6], and M6 [7]); two-tower multimodal pre-training (e.g., CLIP [8] and ALIGN [9]); our BriVL.

a

Method	w/ SA Layers?	Contrastive Algorithm	Image-to-Text Retrieval Recall@1	Image-to-Text Retrieval Recall@5	Image-to-Text Retrieval Recall@10	Text-to-Image Retrieval Recall@1	Text-to-Image Retrieval Recall@5	Text-to-Image Retrieval Recall@10	Recall@SUM
BriVL w/ SimCLR	yes	SimCLR-based	27.50	47.32	55.50	27.10	46.84	55.19	259.45
BriVL w/o SA	no	MoCo-based	28.56	47.98	55.83	28.02	46.86	54.58	261.83
BriVL	yes	MoCo-based	**29.82**	**49.13**	**56.47**	**29.28**	**48.12**	**55.84**	**268.66**

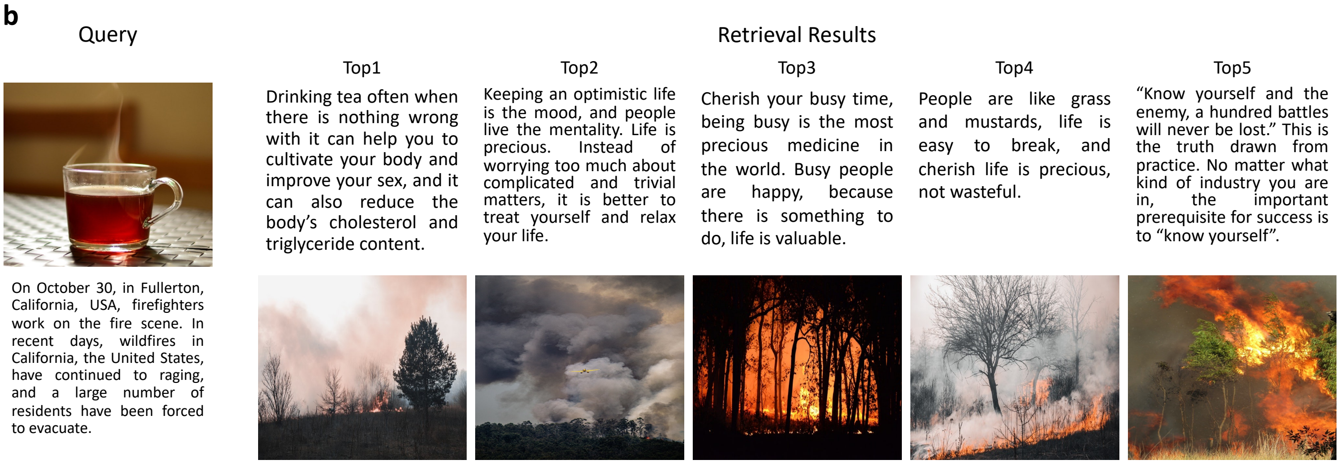

Fig. S2: **Quantitative and qualitative results with 22M training data.** **a**. Ablative results (%) of BriVL on the 11K test set. **b**. Cross-modal retrieval examples of our standard BriVL model (note that images were taken from the Pexels website for illustration). Texts are translated into English for representation clarity. Highest results are highlighted in bold.

Architecture Overview

In Fig. S1a, we present the schematic illustration of our proposed BriVL model for large-scale multimodal pre-training. In Fig. S1b, we show the network details of our image encoder in BriVL. In Fig. S1c, existing architectures for pre-training are illustrated. Please see Methods of the main manuscript for more information.

Ablation Study

To show the contributions of the self-attention (SA) layers used in both encoders and the cross-modal contrastive loss based on MoCo [10], we conduct experiments by whether using SA layers and adopting alternative contrastive losses. Since it is too costly to conduct the ablation study on our full WSCD dataset (of 650M image-text pairs), we only use 22M image-text pairs for training and another 11K for evaluation. We provide the same 9 machines (each has 8 NVIDIA A100 GPUs) for all ablation experiments. The compared methods are as follows: (1) BriVL w/ BYOL: We replace the MoCo-based cross-modal contrastive algorithm in our standard BriVL model with BYOL [11], which does not need any negative samples and only focuses on matching the positive ones. (2) BriVL w/ SimCLR: We replace the MoCo-based cross-modal contrastive algorithm with SimCLR [12], which does not have momentum encoders or negative sample queues, and computes the InfoNCE loss [13] within each batch. In other words, the mini-batch size decides the number of negative samples for each positive image-text pair. Note that CLIP [8] and ALIGN [9] both adopt SimCLR-based contrastive loss. (3) BriVL w/o SA: We discard the SA layers from both image and text encoders comparing to our standard BriVL. (4) BriVL: Our standard model with SA layers and MoCo-based cross-modal contrastive algorithm. All methods are trained for 6 epochs.

The ablative results are shown in the table of Fig. S2a. Note that we do not report the results of BriVL w/ BYOL because no matter how we try (e.g., tuning the hyper-parameters and implementing in different ways), the model always collapses. One possible reason is that BYOL only works under single-modal scenarios and it is essential to include negative samples under multimodal scenarios. Moreover, since SimCLR has neither momentum encoders nor negative sample queues, its mini-batch size N_b can be larger than that of standard BriVL with the same computational resources. Concretely, the total batch size is 2,160 for SimCLR and 1,728 for standard BriVL which additionally maintains two negative sample queues with the size of 10,368 (since momentum encoders and negative sample queues do not produce gradients, they take up little GPU memory). However, we can then see from the table that BriVL w/ SimCLR performs worse than our standard BriVL (based on MoCo) for all 7 evaluation metrics. This indicates

a

Method	Question Type						
	What	Where	When	Who	Why	How	Overall
BriVL-en (direct training)	76.74	77.13	78.12	76.21	76.45	77.55	76.91
BriVL-en (pre-train & zero-shot)	52.40	53.24	53.11	53.29	52.34	52.98	52.75
BriVL-en (pre-train & finetune)	**81.20**	**81.69**	**81.36**	**80.45**	**80.64**	**81.75**	**81.26**

b

Method	BLEU @4	METEOR	CIDEr	SPICE	ROUGE-L
BriVL-en (direct training)	18.87	14.44	37.06	12.71	44.01
BriVL-en (pre-train & finetune)	**20.18**	**21.90**	**44.47**	**15.54**	**45.85**

c

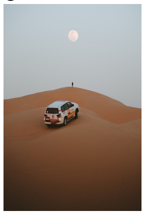

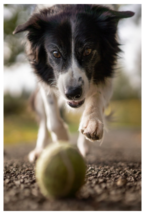

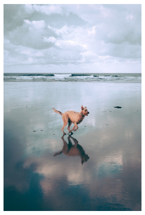

Fig. S3: **Results on two English downstream tasks. a**. Visual question answering results on Visual7W. Overall accuracies (%) along with results on each question type are reported. **b**. Image captioning results (%) on the test split of Flickr30K. **c**. Image captioning examples of our BriVL-en model regarding whether it is pre-trained. Highest results are highlighted in bold.

the importance of large number of negative samples in multimodal pre-training and the advantage of our standard BriVL when large batch size is not feasible. Our model design thus hopefully helps those researchers with limited GPU resources for multimodal pre-training. Besides, comparing to our standard BriVL, discarding the SA layers (i.e., BriVL w/o SA) also leads to performance drop, which validates the effectiveness of this module.

Furthermore, in Fig. S2b, we present two cross-modal retrieval examples with our standard BriVL model trained on the 22M data. The query image for text retrieval and the candidate images for image retrieval are all taken from the Pexels website (`https://www.pexels.com/`), while the texts are picked from the 11K test set. We can observe that the returned top-5 texts for the query image containing a cup of tea are all philosophical sentences, validating the effectiveness of training BriVL over weak semantic correlation data. Further, for the text query in the second row, our BriVL also does a great job in finding the matched images.

Results on English Tasks

In this section, we pre-train our proposed model on a well-known English dataset and name it as BriVL-en. Concretely, the pre-training English dataset consists of 4 publicly-available image captioning datasets. (1) **MSCOCO** [14]: We only use the training split of MSCOCO, which contains 113,287 images (each image has 5 text captions). (2) **Flickr30K** [15]: We use the training set of Flickr30K, which contains 29,783 images (each image also has 5 text captions). (3) **SBU Captioned Photo Dataset (SBU)** [16]: We collect SBU by the provided urls and obtain around 867K image-text pairs. We use the whole SBU dataset. (4) **Conceptual Captions (CC)** [17]: We also collect and use the whole CC dataset, which contains around 3M image-text pairs. As a result, the total size of the pre-training English dataset is around 4M.

In the next two subsections, we conduct experiments on two downstream tasks (i.e., visual question answering and image captioning) to show the potential use of our English model BriVL-en. Importantly, the obtained similar results indicate that our model indeed provides a feasible solution closer to AGI beyond specific languages.

Visual Question Answering. We first conduct visual question answering (VQA) experiments on the Visual7W dataset [18] with three BriVL-en variations. The dataset split is the same as that for Chinese VQA experiments in the main paper, but this time we do not need to translate the texts. In the table of Fig. S3a, we report the overall accuracies on the test set of Visual7W, as well as the results on each question type. We can make the following observations: (1) "BriVL-en (pre-train & zero-shot)" performs much worse than "BriVL-en (direct training)". This is mainly because the VQA task has a large domain gap to our pre-training task and the data distributions are also totally different. (2) "BriVL-en (pre-train & finetune)" outperforms "BriVL-en (direct training)" by large margins for all evaluation metrics, indicating the effectiveness of the pre-trained model and also the importance of finetuning when the data distribution of the downstream task is different with that of the pre-training data.

Image Captioning. Image captioning aims to generate descriptions for given images. In the table of Fig. S3b, we report the results on the test split of Flickr30K [15] (1K images) w.r.t. five commonly-used evaluation metrics in the field of image captioning. Since an additional Transformer [19] decoder is needed to generate texts, we cannot conduct

zero-shot experiments. We can see that "BriVL-en (pre-train & finetune)" outperforms "BriVL-en (direct training)" for all metrics, again validating the effectiveness of pre-training.

Furthermore, we present three image captioning examples in Fig. S3c. For the first image, "BriVL-en (pre-train & finetune)" points out that the car is white instead of red, and is parked in the desert rather than driving down a dirt road. Actually it is hard to tell whether the car is driving or parked. But as we see a man standing out there, it is highly possible that the car is parked (which is commonsensical). For the second image, "BriVL-en (pre-train & finetune)" knows that the dog is catching the ball rather than "running on the grass with a ball in its mouth". Here, our pre-trained BriVL-en describes the action of the dog running towards the ball as "catching a ball", which is impressive. For the third image, despite the reflection under the dog, "BriVL-en (pre-train & finetune)" sees that the dog is running on a beach rather than across the water. This also shows the hint of common sense because running across the water is illogical and the dog is most likely to be on a beach considering the distant waves.

Image Sources

Except the images that are owned by us, all others used in both the main manuscript and the supplementary note are taken from the Pexels website (https://www.pexels.com), which provides free stock photos and allows users to download for free use (see its license page "https://www.pexels.com/license/" for more information). We list all images that are taken from the public (i.e., the Pexels website) in Table S1.

Table S1: The sources of images used in this work.

	**Image**	**Source (URL)**
1	The cake image in Fig. 1b.	https://www.pexels.com/photo/a-close-up-shot-of-a-cake-with-a-candle-on-top-8015277/
2	The "baseball field" image at the top of Fig. 4c.	https://www.pexels.com/photo/city-road-landscape-flying-9739479/
3	The first image (from left) in Fig. 6c.	https://www.pexels.com/photo/selective-focus-photography-of-train-610683/
4	The second image (from left) in Fig. 6c.	https://www.pexels.com/photo/photo-of-horses-grazing-in-grass-field-2050425/
5	The third image (from left) in Fig. 6c.	https://images.pexels.com/photos/752882/pexels-photo-752882.jpeg
6	The fourth image (from left) in Fig. 6c.	https://www.pexels.com/photo/vehicles-stop-on-red-light-771184/
7	The picnic image in Fig. S1a and Fig. S1b.	https://www.pexels.com/photo/food-platters-on-picnic-blanket-5076436/
8	The upper-left middle image in Fig. S1a.	https://www.pexels.com/photo/white-and-blue-floral-table-lamp-1793037/
9	The upper-left back image in Fig. S1a.	https://www.pexels.com/photo/green-christmas-tree-with-baubles-6139342/
10	The tea cup image in Fig. S2b.	https://www.pexels.com/photo/teacup-with-tea-905485/
11	The first image (from left) in the second row of Fig. S2b.	https://www.pexels.com/photo/bushfire-4070651/
12	The second image (from left) in the second row of Fig. S2b.	https://www.pexels.com/photo/yellow-plane-flying-over-a-forest-fire-4902033/
13	The third image (from left) in the second row of Fig. S2b.	https://www.pexels.com/photo/photo-of-burning-forest-4621457/
14	The fourth image (from left) in the second row of Fig. S2b.	https://www.pexels.com/photo/forest-fire-4070727/
15	The fifth image (from left) in the second row of Fig. S2b.	https://www.pexels.com/photo/blazing-fire-in-the-forest-4636324/
16	The left image in Fig. S3c.	https://www.pexels.com/photo/car-on-a-dessert-4318822/
17	The middle image in Fig. S3c.	https://www.pexels.com/photo/tilt-shot-photo-of-dog-chasing-the-ball-1562983/
18	The right image in Fig. S3c.	https://www.pexels.com/photo/dog-running-at-the-beach-2906033/

Supplementary References

[1] Radford, A., Narasimhan, K., Salimans, T. & Sutskever, I. Improving language understanding by generative pre-training. *OpenAI Blog* (2018).

[2] Devlin, J., Chang, M.-W., Lee, K. & Toutanova, K. Bert: Pre-training of deep bidirectional transformers for language understanding. *arXiv preprint arXiv:1810.04805* (2018).

[3] Kolesnikov, A. *et al.* Big transfer (bit): General visual representation learning. In *European Conference on Computer Vision*, 491–507 (2020).

[4] Dosovitskiy, A. *et al.* An image is worth 16x16 words: Transformers for image recognition at scale. In *International Conference on Learning Representations* (2021).

[5] Chen, Y.-C. *et al.* Uniter: Universal image-text representation learning. In *European Conference on Computer Vision*, 104–120 (2020).

[6] Li, X. *et al.* Oscar: Object-semantics aligned pre-training for vision-language tasks. In *European Conference on Computer Vision*, 121–137 (2020).

[7] Lin, J. *et al.* M6: A chinese multimodal pretrainer. *arXiv preprint arXiv:2103.00823* (2021).

[8] Radford, A. *et al.* Learning transferable visual models from natural language supervision. In *International Conference on Machine Learning*, 8748–8763 (2021).

[9] Jia, C. *et al.* Scaling up visual and vision-language representation learning with noisy text supervision. In *International Conference on Machine Learning*, 4904–4916 (2021).

[10] He, K., Fan, H., Wu, Y., Xie, S. & Girshick, R. Momentum contrast for unsupervised visual representation learning. In *IEEE Conference on Computer Vision and Pattern Recognition*, 9729–9738 (2020).

[11] Grill, J.-B. *et al.* Bootstrap your own latent - a new approach to self-supervised learning. In *Advances in Neural Information Processing Systems*, 21271–21284 (2020).

[12] Chen, T., Kornblith, S., Norouzi, M. & Hinton, G. A simple framework for contrastive learning of visual representations. In *International Conference on Machine Learning*, 1597–1607 (2020).

[13] Oord, A. v. d., Li, Y. & Vinyals, O. Representation learning with contrastive predictive coding. *arXiv preprint arXiv:1807.03748* (2018).

[14] Lin, T.-Y. *et al.* Microsoft coco: Common objects in context. In *European Conference on Computer Vision*, 740–755 (2014).

[15] Young, P., Lai, A., Hodosh, M. & Hockenmaier, J. From image descriptions to visual denotations: New similarity metrics for semantic inference over event descriptions. *Transactions of the Association for Computational Linguistics* **2**, 67–78 (2014).

[16] Ordonez, V., Kulkarni, G. & Berg, T. L. Im2text: Describing images using 1 million captioned photographs. In *Advances in Neural Information Processing Systems*, 1143–1151 (2011).

[17] Sharma, P., Ding, N., Goodman, S. & Soricut, R. Conceptual captions: A cleaned, hypernymed, image alt-text dataset for automatic image captioning. In *Proceedings of the 56th Annual Meeting of the Association for Computational Linguistics*, 2556–2565 (2018).

[18] Zhu, Y., Groth, O., Bernstein, M. S. & Fei-Fei, L. Visual7w: Grounded question answering in images. In *IEEE Conference on Computer Vision and Pattern Recognition*, 4995–5004 (2016).

[19] Vaswani, A. *et al.* Attention is all you need. In *Advances in Neural Information Processing Systems*, 5998–6008 (2017).
